# Continuous degradation of phenanthrene in cloud point system by reuse of *Sphingomonas polyaromaticivorans* cells

**DOI:** 10.1186/s13568-019-0736-2

**Published:** 2019-01-19

**Authors:** Tao Pan, Rennv Wang, Kun Xiao, Wei Ye, Wei Dong, Meiying Xu

**Affiliations:** 1 Jiangxi Key Laboratory of Mining & Metallurgy Environmental Pollution Control, and School of Resource and Environmental Engineering, Jiangxi University of Science and Technology, Ganzhou, 341000 China; 2 State Key Laboratory of Applied Microbiology Southern China, Guangdong Institute of Microbiology, Guangdong, 510070 China

**Keywords:** Cloud point system, Phenanthrene, Biodegradation, Reuse, *Sphingomonas polyaromaticivorans*

## Abstract

Extractive biodegradation of phenanthrene by *Sphingomonas polyaromaticivorans* was previously carried out in cloud point system (CPS). In this study, we explored the possibility of further increasing the efficiency of the culture by repeatedly reusing cells and the system for biodegradation. Three different recycling strategies were adopted. In reuse of cells plus CPS, cells were reused for 3 times while maintaining high degradation rates (> 90%). Thereafter, the accumulation of metabolites in the dilute phase resulted in a decrease in cell viability. This inhibition was avoided in recycling the cells plus coacervate phase by replacing the dilute phase with fresh Medium. However, due to the slow adaptation of the cells to the new degradation environment and the reduction in the volume of the coacervate phase, the cells were only reused twice and their activity decreased. However, the same long degradation cycle (5 days) as the reuse of cells plus coacervate phase reduced the overall degradation efficiency of phenanthrene. Finally, a combined strategy of 3 times of cells plus CPS reuse and individual cells reuse once was employed and run for two cycles. 3 rounds of reuse of cells plus CPS improved cells utilization and phenanthrene degradation efficiency. Then, the subsequent round of reuse of cells alone relieved the effect of increasing metabolites on cell viability. This study provides a potential application for reusing cells to continuously degrade phenanthrene in soil and water in CPS.

**Electronic supplementary material:**

The online version of this article (10.1186/s13568-019-0736-2) contains supplementary material, which is available to authorized users.

## Introduction

Polycyclic aromatic hydrocarbons (PAHs) are the most important class of pollutants in the environment (Trellu et al. 2016). They are extremely harmful due to the high bioaccumulation toxicity (Alharbi et al. 2018). Biodegradation is the main method for repairing PAHs (Haritash and Kaushik 2009; Dangi et al. 2019). However, the bioavailability of PAHs is low, because PAHs are highly adsorbed and enriched in soil, sediment, and suspended particles in the environment (Maletić et al. 2019; Alegbeleye et al. 2017). Therefore, how to improve bioavailability has become a key issue in the biodegradation of PAHs (Johnsen and Karlson 2007; Guo et al. 2016).

The addition of a surfactant to form a solubilizing system is a commonly used bioavailability strengthening method (Miller 1995). This has been used to promote microbial degradation of PAHs and has been reported many times (Lamichhane et al. 2017). However, the widespread use of surfactants is still controversial (Liu et al. 2017). Laha and Luthy first discovered that the nonionic surfactants C12E4, C8PE9.5 and C9PE10.5 inhibited the degradation of phenanthrene at concentrations above the critical micelle concentration (CMC) (Laha and Luthy 1991). Li et al. proposed to use biosurfactants with better biocompatibility instead of chemical surfactants, which may be beneficial to relieve these inhibition effects (Li and Chen 2009). However, Xiao et al. found that rhamnolipid slightly higher than CMC concentration (0.02–0.5 g/L) can promote the degradation of dichlorodiphenyltrichloroethane by white rot fungus *Phlebia lindtneri* GB1027, but excessive concentration (> 1.0 g/L) will produce toxic cells (Xiao et al. 2014). Micelle solutions formed by low concentrations of surfactants enhanced the phase transfer of contaminants but inhibited bacterial adhesion to soil contaminants (Ortega-Calvo and Alexander 1994; Stelmack et al. 1999). In our previous studies, it was found that during the degradation of phenanthrene by *Sphingomonas polyaromaticivorans*, the micellar system with low surfactants concentration inhibited cell metabolism, while the cloud point system (CPS) with high surfactants concentration played a reinforcing role (Pan et al. 2017b). The so-called CPS is a two-phase system in which a nonionic surfactant aqueous solution is formed by phase separation at a certain temperature (Wang et al. 2008). The surfactant-rich phase is coacervate phase and the phase with a low surfactant concentration is dilute phase. The phase separation temperature is cloud point. Compared to the micellar system, the coacervate phase of the CPS relieved the substrate and product inhibition of the contaminants, thus enhancing biodegradation (Pan et al. 2017b).

In order to improve the efficiency of biodegradation of pollutants, the reuse of degrading bacteria cells is a feasible solution. Reuse of *Sphingomonas* sp. CDH-7 cells achieved continuous degradation of carbazole (Nakagawa et al. 2002). The restore of cells in buffer with MgCl_2_ enhanced their degradation activity. In CPS, cells recycling has also been performed in biotransformation. Wang et al. reused resting cells of *Mycobacterium* for 3 times to produce androsta-diene-dione from phytosterol in CPS (Wang et al. 2006). However, in CPS, the reuse of cells in biodegradation has not been tried. The metabolic activity of the cells was well maintained in the CPS (Pan et al. 2017b). In order to exploit the metabolic potential of these cells, we decided to conduct cell recycling experiments to perform continuous degradation of phenanthrene. Three recycling protocols were tested, including cells plus CPS (1), cells plus coacervate phase (2), and cells alone (3). Finally, we present the best solution for the recovery and reuse of *S. polyaromaticivorans* cells in the CPS to continuously degrade phenanthrene.

## Materials and methods

### Chemical reagents

Phenanthrene, Tergitol TMN-3, and Brij 30 were purchased from Sigma-Aldrich (St. Louis, Missouri, United States). Acetonitrile was of high performance liquid chromatography (HPLC) grade. All other chemicals were of analytical grade.

### Microorganism

The strain used in the experiment was *S. polyaromaticivorans*, deposited in China Center of Industrial Culture Collection (CICC), with an Accession Number of CICC No. 10894 (Pan et al. 2016). The strain was kept on Luria–Bertani (LB) agar medium (tryptone 10 g, yeast extract 5 g, NaCl 10 g, and agar 20 g, dissolved in 1 L of double distilled water and adjusted pH to 7) at 4 °C.

### Preparation of cells

Single colony was picked from well-grooved solid medium and activated in LB medium for two times. Subsequently, it was inoculated into 30 mL LB medium in a 150-mL flask and cultured at 30 °C, 160 rpm for 18 h. The cells were then collected by cryo-centrifugation, washed twice with 50 mmol/L Tris–HCl (pH 7.1), and resuspended in mineral salt medium (MSM) for reserve.

### Biodegradation and culture conditions

Degradation media and culture conditions refer to our previously published study (Pan et al. 2016). MSM was used for the biodegradation of phenanthrene, and its composition was (NH_4_)_2_SO_4_ 1.0 g, Na_2_HPO_4_ 0.8 g, KH_2_PO_4_ 0.2 g, MgSO_4_·7H_2_O 0.2 g, FeCl_3_·3H_2_O 0.005 g, CaCl_2_·2H_2_O 0.1 g, (NH_4_)_6_Mo_7_O_24_4H_2_O 0.001 g, and 1 L of double distilled water. The concentration of the substrate phenanthrene was set at 400 mg/L. The CPS was formed by adding 20 g/L of mixed surfactants (Brij 30:TMN-3, 1:1) to the MSM. A 20 g/L of wet cells was inoculated into 30 mL MSM in a 150-mL flask for biodegradation of phenanthrene. The degradation experiment was performed at 30 °C and 160 rpm.

### Cells recycling protocols

As mentioned earlier, three sets of cells recycling protocols were tested. For reuse of cells plus CPS (1), phenanthrene was added directly to the original system without any changes of medium; For reuse of cells plus coacervate phase (2), we discarded dilute phase of CPS and then replenish the same volume of MSM. Then, phenanthrene was added for another round of degradation experiment; For reuse of the cells alone (3), cells harvested by centrifugation were washed 3 times with MSM and then inoculated into a fresh CPS with newly added phenanthrene.

### Analysis methods

The metabolites of phenanthrene was a carotenoid pigment was produced during the biodegradation by *S. polyaromaticivorans*. According to full-wavelength scanning analysis by UV–visible spectrophotometer (UV1750, SHIMADZU, Japan), its maximum absorbance was at 474 nm. Therefore, the content of the metabolite was represented by its absorbance at 474 nm.

Phenanthrene was detected by HPLC Agilent_1260 equipped with Agilent G1314BC automated variable-wavelength UV–VIS detector and using an Agilent Hypersil C18 column (5 μm, 150 mm × 4.6 mm). Phenanthrene was detected at 254 nm with acetonitrile:water (80:20) as the mobile phase (1.0 mL/min).

## Results

### The metabolites of phenanthrene in cells and CPS

**Fig. 1:**
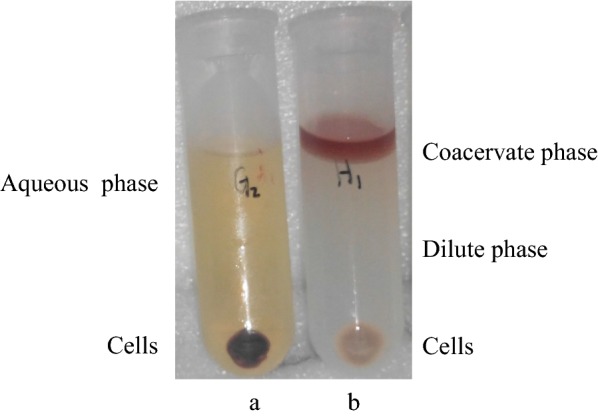
The metabolites of phenanthrene in cells and CPS. **a** Control system; **b** CPS. The biodegradation of phenanthrene was carried out in 30 mL MSM in 150-mL shaking flask at 30 °C and 160 rpm for 5 days in control and CPS by *S. polyaromaticivorans*, respectively. Then, cells were harvested by centrifuge at 4 °C and 8000 rpm in the control (**a**) or CPS (**b**). The distribution of metabolite in both systems was shown in the figure

The biodegradation of phenanthrene in CPS and control system without surfactants was carried out, respectively. The metabolite of phenanthrene in cells and its partition in control and CPS were displayed in Fig. 1. In the control system, most of the pigment produced was adsorbed on the cells, leaving a small part dissolved in the aqueous phase (Fig. 1a). However, in the CPS, the pigment in the cells was extracted into the coacervate phase, which resulted in a lighter color of cells and colorless dilute phase (Fig. 1b).

### Effect of cells age on biodegradation

**Fig. 2:**
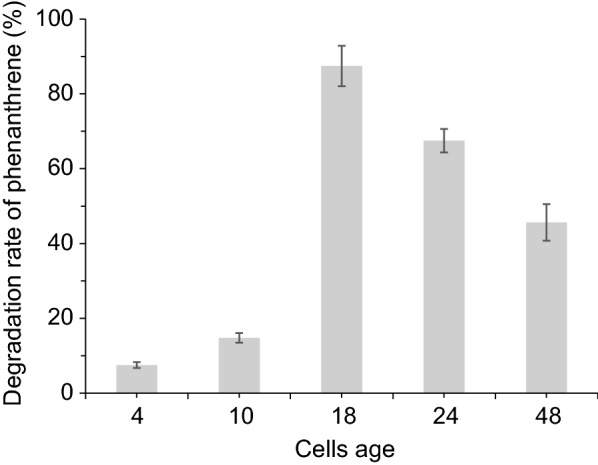
Effect of cells age on biodegradation of phenanthrene. *S. polyaromaticivorans* was inoculated into 30 LB medium in a 150-mL flask and cultured at 30 °C and 160 rpm. Cells were collected after 4, 10, 18, 24, and 48 h of culture, respectively, and then inoculated in CPS to degrade phenanthrene for 5 days

The effect of cells age on phenanthrene biodegradation was performed in CPS for 5 days (Fig. 2). The degradation rate of phenanthrene represented the metabolic activity of cells at different growth stages. Very small amounts of phenanthrene were degraded by cells in the lag (4 h) and early logarithmic growth (10 h) stages. As the cell culture time increased, more phenanthrene was degraded. This indicated that the cellular metabolic activity was gradually enhanced. When the cells age was at 18 h, the degradation rate was the highest, and then gradually decreased.

### Reuse of cells plus CPS

**Fig. 3:**
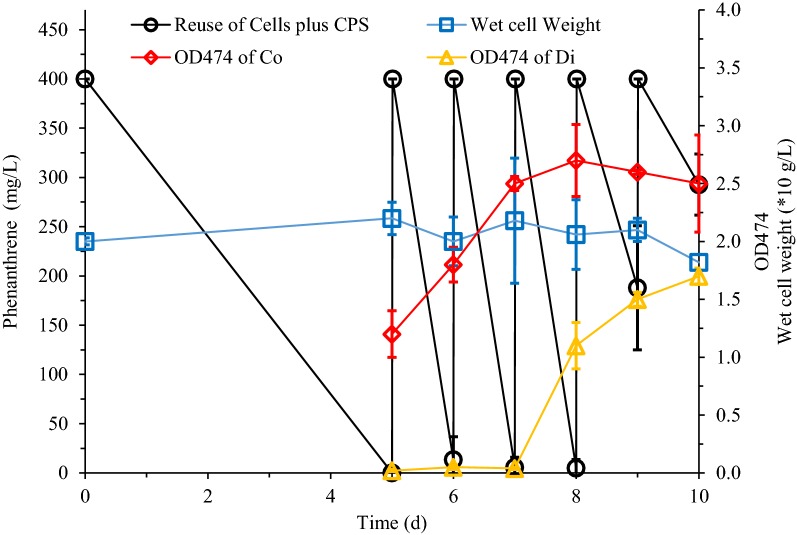
Reuse of cells plus CPS for phenanthrene biodegradation. *S. polyaromaticivorans* was inoculated into 30 LB medium in a 150-mL flask and cultured at 30 °C and 160 rpm for 18 h. Cells were collected and then inoculated in CPS to degrade phenanthrene for 5 days. Subsequently, 400 mg/L of phenanthrene was added every other day. Co: coacervate phases; Di: dilute phase

Reuse of cells plus CPS for phenanthrene biodegradation was carried out for 5 times in succession (Fig. 3). The cells obtained from the LB medium initially took 5 days to adapt to the degradation environment. Subsequently, within 1 day, phenanthrene added per round could be degraded more than 90%. This high degradation rate maintained three reuse cycles. Then, the metabolic capacity of the cells began to decline, and the degradation rate was less than 50%. Moreover, in the first four rounds of degradation, we observed that the color of the cloud layer coacervate phase gradually increased with the increasing OD474, while the color of the dilute phase hardly changed. In the subsequent two rounds, the absorbance of the coacervate phase remained constant, while the absorbance of the dilute phase began to rise sharply. During the entire recycling experiment period, the amount of cells remained substantially constant except for a small increase in the first 5 days.

### Reuse of cells plus coacervate phase

For the reuse of cells and coacervate phases, the degradation cycle for each round is up to 5 days. Even more unfortunately, the degradation activity of the cells only reused twice, was greatly reduced. The most obvious change was the volume of the coacervate phase, which was dropped from 2 mL to only 0.2 mL after three times of reuse. This also resulted in a large amount of secondary metabolites produced by cell degradation remaining in the dilute phase. This was also reflected in absorbance slowing down in the coacervate phase and sharply rising in the dilute phase. Meanwhile, the amount of cells did not continue to increase due to the addition of fresh MSM medium, and began to decline after two rounds.

### Reuse of cells alone

The continuous degradation of phenanthrene was carried out by cells recovered alone in each round (Fig. 5). The volume of the coacervate phase and the OD474 absorbance of both phases were almost unchanged, since the culture system was replaced each time (dates not shown). Similar to the reuse of cells and coacervates, cells also need at least 5 days to achieve a degradation rate of more than 90%. Fortunately, the cells maintained a steady growth after each round of degradation, and maintained stable phenanthrene degradation ability after five rounds.

### Combination of cell reusing protocols

Based on the advantages and disadvantages of the above three recycling schemes, three rounds of cells plus CPS reuse and one round of cells reuse were combined and cycled 4 times, as shown in Fig. 6. For reuse of cells plus CPS shown in Fig. 3, the cell metabolic activity was reduced after four rounds. To avoid this, the reuse of cells plus CPS in each cycle was only executed for 3 times in this joint reuse experiment. In each cycle, after three rounds of reuse of cells plus CPS, the used CPS will be replaced with fresh ones and only the cells will be reused. In the first and second cycles, the continuous degradation of phenanthrene by the cells was still higher than 90%. Synchronously, apparent cell growth was also observed with the newly replaced medium each time. However, the amount and metabolic activity of cells began to decline from the third cycle. Especially in the fourth cycle, the filamentous material formed by the cell debris was already clearly visible in the solution (Additional file 1: Figure S1). At the end of the experiment, the degradation rate of phenanthrene has fallen below 50%.

## Discussion

In previous studies, we have reported the extractive biodegradation of PAHs in CPS (Pan et al. 2016). As a buffer pool for the substrate, coacervate phase of CPS increased the bioavailability of the substrate and prevented its inhibition through the sustained release of the substrate (Pan et al. 2016, 2017b). In the present study, we first discovered that product inhibition was removed in biodegradation, as CPS extracted toxic metabolites from cells to the coacervate phase (Fig. 1). Although in biotransformation, studies have been reported that the coacervate phase acts as a reservoir for the product to relieve its feedback inhibition (Wang et al. 2008). However, to our knowledge, in biodegradation, there is no relevant report.

The phenanthrene degradation response of cells inoculated in the CPS at different growth stages was tested. Young cells were in a period of vigorous growth but almost incapable of degrading phenanthrene (Fig. 2), as their fragile cell membranes were more susceptible to surfactants (Zhang et al. 2013). Older cells were also very inefficient in the degradation of phenanthrene due to aging, autolysis, and intracellular enzyme release (Ghosal et al. 2016; Ventura-Camargo et al. 2016). Cells harvested at 18 h showed the best degradation efficiency, indicating that they had higher cell viability at this time. This also explained why cells used for inoculation were not cultured beyond the stationary phase in most biodegradation tests of polycyclic aromatic hydrocarbon (Rabodonirina et al. 2018).

**Fig. 4:**
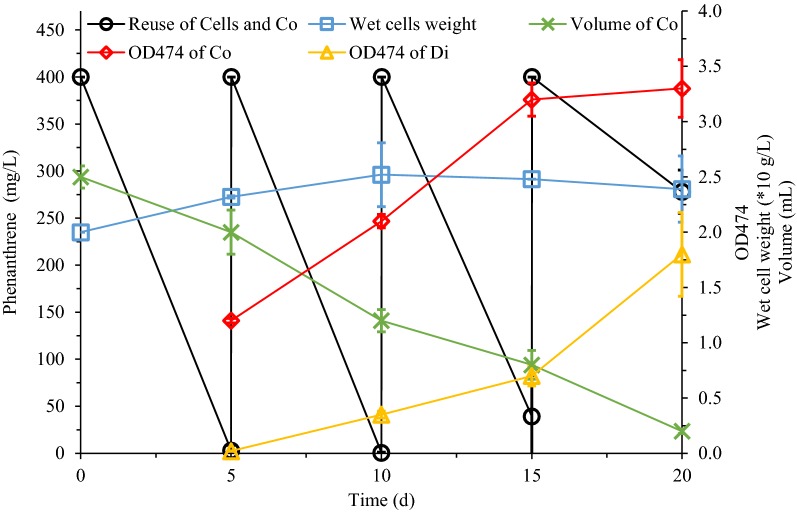
Reuse of cells plus coacervate phase for phenanthrene biodegradation. *S. polyaromaticivorans* was inoculated into 30 mL LB medium in a 150-mL flask and cultured at 30 °C and 160 rpm for 18 h. Cells were collected and then inoculated in CPS to degrade phenanthrene for 5 days. Subsequently, the dilute phase of CPS was discarded and 400 mg/L of phenanthrene was added every 5 days. Co: coacervate phases; Di: dilute phase

**Fig. 5:**
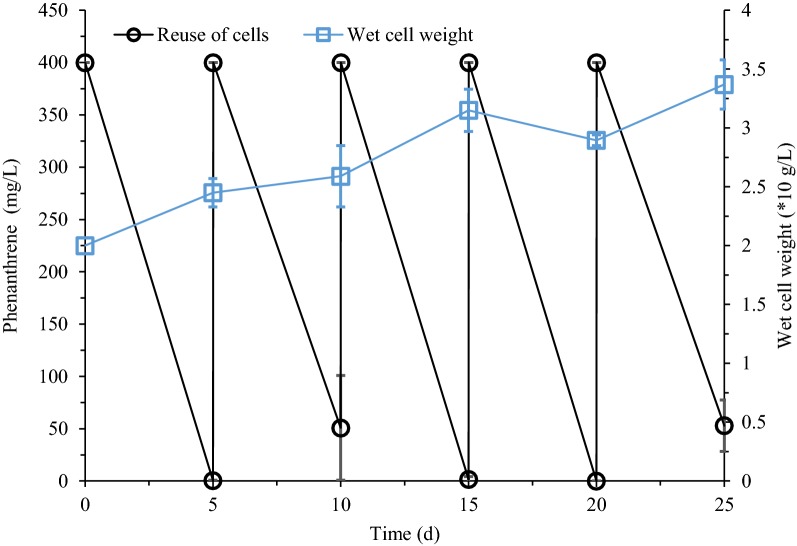
Reuse of cells alone for phenanthrene biodegradation. *S. polyaromaticivorans* was inoculated into 30 mL LB medium in a 150-mL flask and cultured at 30 °C and 160 rpm for 18 h. Cells were collected and then inoculated in CPS to degrade phenanthrene for 5 days. Subsequently, cells were recovered every 5 days and added to a fresh CPS containing 400 mg/L phenanthrene

In order to exploit the phenanthrene degradation potential of cells, we tested various continuous reuse strategies of cells in the cloud point system in this work. It was common to use resting cells to degrade contaminants (Jia et al. 2006). For reuse of cells plus CPS, the almost constant cell content indicated that during this process, the cells entered a resting state (Fig. 3) due to the depletion of other nutrients such as nitrogen sources (Acedos et al. 2018). Limiting the supply of nitrogen sources was a common method of making resting cells (Wang et al. 2016). However, in other repeated experiments (Figs. 4, 5), due to the release of the nitrogen source restriction, the cells grew significantly without remaining in a resting state. This growth was obvious but phenanthrene degradation was slow (5 days), because in the reuse of cells plus coacervate phase (Fig. 4) and the reuse of cells alone (Fig. 5), the cells need to adapt to the new culture environment with the replaced fresh MSM (Peng et al. 2008). In the reuse of cells and CPS (Fig. 3), cells that remained in a resting state were more efficient in degradation of phenanthrene (1 day).

The number of cells reuses is closely related to the state of the coacervate phase of CPS. For reuse of cells plus CPS (Fig. 3), after three rounds of reuse, the extraction of the coacervate relative to the accumulated metabolites was saturated and more metabolites remained in the cells. Therefore, the last two rounds of cellular metabolic activity have been greatly affected, and the degradation rate of phenanthrene is greatly reduced. In Fig. 4, the replacement of the dilute phase supplemented the cells with nutrients other than the carbon source. Although the replacement of the dilute phase alleviated the cytotoxic effect due to the residue of the metabolite in the reuse of cells plus CPS (Fig. 3). However, a rapid decreased in the volume of the coacervate phase was observed. The CPS is formed by phase separation of nonionic surfactants in an aqueous solution (Racheva et al. 2018). Each time in Fig. 4, once the dilute phase was replaced by MSM, a portion of the surfactant of the coacervate phase was replenished into the MSM according to the phase separation principle, which was similar to coacervate phase reuse in the biodegradation of diphenyl ether in our previous work (Pan et al. 2017a). After three cycles, the volume of the coacervate phase was negligible. The solubilization capacity of the coacervate phase relative to the metabolites was also minimized, which ultimately affected the metabolism of the cells to phenanthrene. Figure 5 showed the situation where only cells were recovered and reused, which was also the strategy used in most cell recycling studies (Nakagawa et al. 2002). As a new CPS with MSM and surfactants being replaced each time, there was no significant change in the distribution of metabolites in the two phases per cycle and the cells grew continuously. This indicated that cells reuse alone (Fig. 5) was better at maintaining cell viability than reuse of cells plus CPS (Fig. 3) and reuse of cells plus coacervate phase (Fig. 4). Kirimura et al. even used *Sphingomonas* sp. CDH-7 to degrade carbazole for more than nine rounds (Kirimura et al. 1999).

**Fig. 6:**
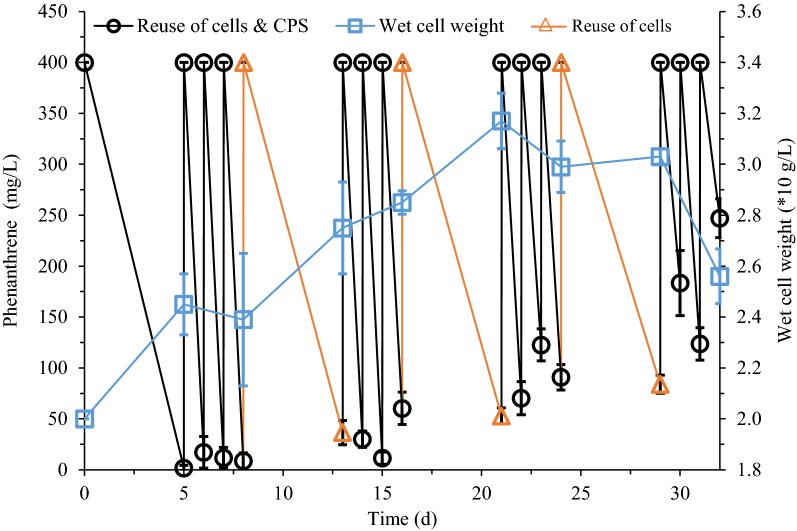
Combined reuse strategy. *S. polyaromaticivorans* was inoculated into 30 mL LB medium in a 150-mL flask and cultured at 30 °C and 160 rpm for 18 h. Cells were collected and then inoculated in CPS to degrade phenanthrene for 5 days. Subsequently, a combined recycling strategy concluding three reuses of cells plus CPS and one reuse of cells alone was repeated

Finally, a combined strategy of 3 times of cells plus CPS reuse and individual cells reuse once was employed and run for two cycles (Fig. 6). 3 rounds of reuse of cells plus CPS improved cells utilization and phenanthrene degradation efficiency. Then, the subsequent round of reuse of cells alone relieved the effect of increasing metabolites on cell viability. Until the third cycle, the visible cell debris in the medium indicated that the cells have aged and autolyzed, resulting in the subsequent uncompleted phenanthrene degradation (Additional file 1: Figure S1) (Ventura-Camargo et al. 2016).

Extractive biodegradation in CPS provided a useful strategy for the pollution control of polycyclic aromatic hydrocarbons (Pan et al. 2016). Cells retained biological activity in CPS offered the possibility of recycling them (Fig. 1). 3 resulting schemes were tested, namely cells plus CPS (Fig. 3), cells plus coacervate phase (Fig. 4), and cells alone (Fig. 5). A combined reuse strategy consisting of three rounds of cells plus CPS and one round of cells alone was adopted (Fig. 6). This maximized degradation efficiency and saved time and costs in future practical applications.

## Additional file

**Additional file 1: Figure S1.** Combination of cell reusing protocols for the third and fourth cycle.

## Abbreviations

PAHs: polycyclic aromatic hydrocarbons
CMC: critical micelle concentration
CPS: cloud point system
HPLC: high performance liquid chromatography
CICC: China Center of Industrial Culture Collection
LB: Luria–Bertani
MSM: mineral salt medium
