# Supplementary material for: Continuous degradation of phenanthrene in cloud point system by reuse of Sphingomonas polyaromaticivorans cells
Source: AMB Express. 2019 Jan 19;9:8. doi: 10.1186/s13568-019-0736-2 (PMC6339633; doi:10.1186/s13568-019-0736-2)
Supplement: Supplementary file 1 — Additional file 1: Figure S1. Combination of cell reusing protocols for the third and fourth cycle. [file 13568_2019_736_MOESM1_ESM.docx]

AMB Express

Additional file 1 for

**Continuous Degradation of Phenanthrene in Cloud Point System by Reuse of *Sphingomonas polyaromaticivorans* Cells**

**Tao Pan^1,2*^, Rennv Wang^1^, Kun Xiao^1^, Wei Ye^2^, Wei Dong^1^, Meiying Xu^2^**

^*^Correspondence: pttianya@gmail.com; t.pan@jxust.edu.cn

^1^ Jiangxi Key Laboratory of Mining & Metallurgy Environmental Pollution Control, and School of Resource and Environmental Engineering, Jiangxi University of Science and Technology, Ganzhou 341000, China

^2^ State Key Laboratory of Applied Microbiology Southern China, Guangdong Institute of Microbiology, Guangdong 510070, China.

**Legends of Figures**

**Fig. S1** Combination of cell reusing protocols for the third and fourth cycle.

**Fig. S1**


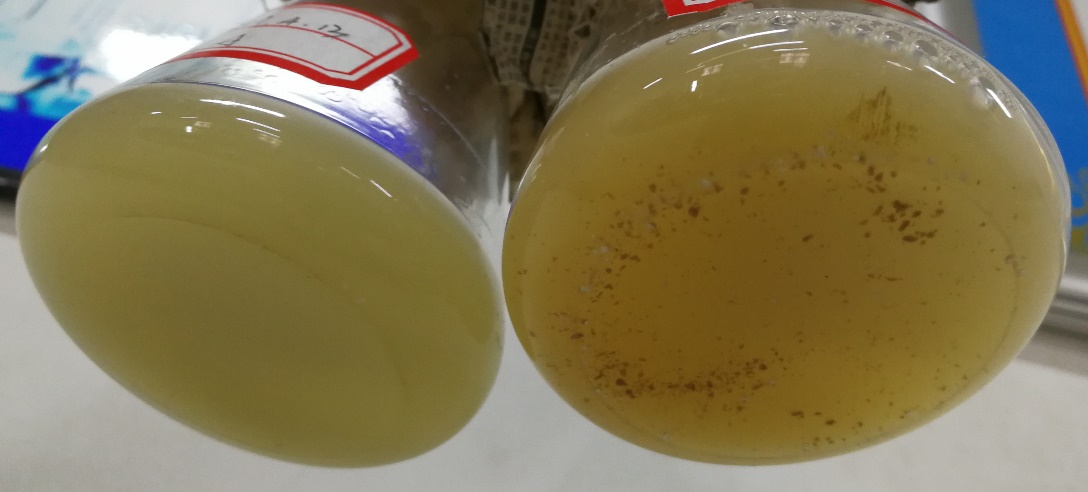


The third cycle The fourth cycle

cell debris
